# 

**Published:** 1879-11

**Authors:** 


### CONTENTS.

COMMUNICATIONS.

Dental Caries. *By S. M. Prothro, of Chatanooga, Tenn*.................... 449
Inductive and Deductive Methods of Study. *By E. G. Betty, D. D. S., Cincinnati*........................................................................ 465
Hydrophobia. *By John J. Caldwell, M. D., Baltimore*......................... 469

SELECTIONS.

Appearance of the Tongue in Disease. *By John A. Henning, M. D., of Red Key, Ind*........................................................................ 478
Analysis of Chlorine........................................................................ 480

EDITORIAL.

Arsenical Poisoning........................................................................ 481
Examination of Candidates for Admission to the Dental Department of the University of Michigan........................................................ 486
Board of Examiners........................................................................ 489
Ohio State Dental Society................................................................ 489
Bibliographical................................................................................ 490
American Health Primers.................................................................. 492
Matrimonial..................................................................................... 492

